# Supplementary material for: MRG15 alternative splicing regulates CDK1 transcriptional activity in mouse cell senescence and myocardial regeneration
Source: Commun Biol. 2025 Jun 7;8:895. doi: 10.1038/s42003-025-08309-z (PMC12145428; doi:10.1038/s42003-025-08309-z)
Supplement: Supplementary file 1 — Supplementary information [file 42003_2025_8309_MOESM1_ESM.pdf]

MRG15 alternative splicing regulates CDK1 transcriptional activity in mouse cell senescence and myocardial regeneration

Yuan Zhang[1,2,3]* Huayu Wang[1]*, Fang Li[1]*, Hui Dai[1], Ye Zhang[1#]

[1]Department of Biochemistry and Molecular Biology, Institute of Basic Medical Sciences, Chinese Academy of Medical Sciences & Peking Union Medical College, Beijing, China

[2]Faculty of Hepato-Pancreato-Biliary Surgery, the First Medical Center, Chinese PLA General Hospital, Institute of Hepatobiliary Surgery of Chinese PLA, Key Laboratory of Digital Hepatobiliary Surgery, PLA, Beijing, China

[3]Department of Neurosurgery, Peking Union Medical College Hospital, Chinese Academy of Medical Sciences and Peking Union Medical College, Beijing, China

# Corresponding authors at: Department of Biochemistry and Molecular Biology, Institute of Basic Medical Sciences, Chinese Academy of Medical Sciences and Peking Union Medical College, Beijing, China

E-mail addresses: yezhang@ibms.pumc.edu.cn

* These authors contribute equally.

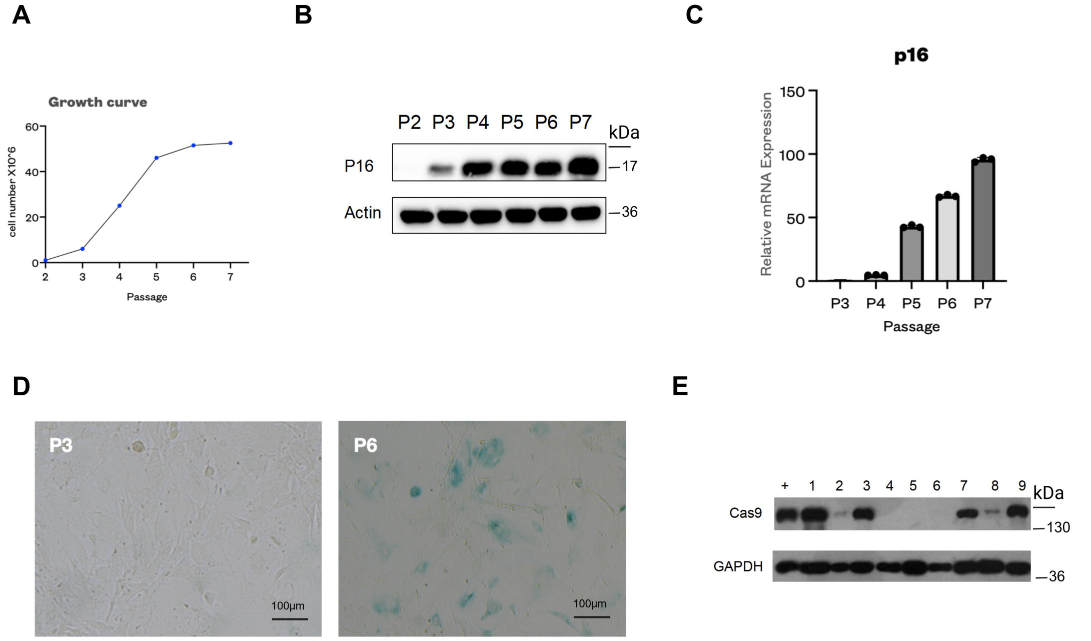

Supplementary Fig. 1: MEF cell passage aging model.

A. The growth curve of MEF cells fused with Cas9, passaged with 3T1, enters a plateau phase from the sixth to seventh generation.

B,C. Protein level of Cas9 in MEF cells and mRNA level increase with passaging.

D. β-galactosidase staining also increases with generation.

E. Cas9 protein expression levels in cells derived from different Cas9 mice (lanes 1 to 9). "+" indicates a Cas9-positive control. GAPDH serves as a loading control.

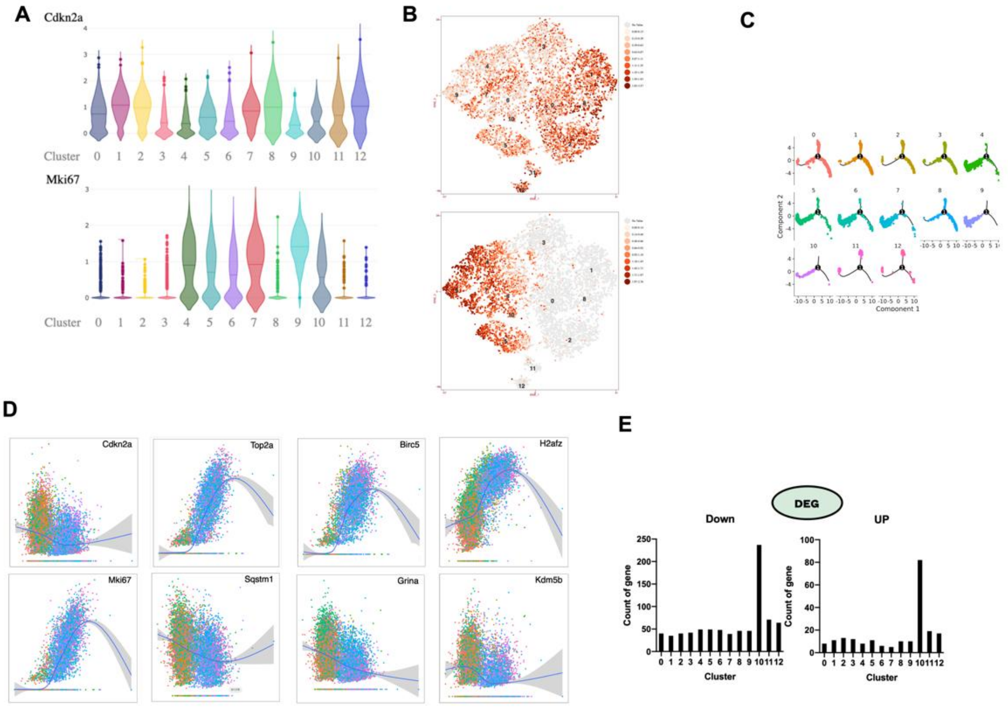

Supplementary Fig. 2: Single-cell sequencing analysis of aging cells.

A. The *Cdkn2a* (*p16*) and *Mki67* levels also show c4, 5, 6, 7, 9, and 10 anti-aging cell clusters.

B. The distribution of *Mki67* and *p16* in the single-cell Tsne map in B is consistent with previous aging classifications.

C. The tendency of c4, 5, 6, 7, 9, and 10 in the pseudo-temporal map C is obviously different from other aging groups. According to the differential genes of c10 cluster cells in experimental group K9 in the PCA map, there are significantly more than other groups, possibly due to the CRISPR-Cas9 bridge. The temporal map also shows that the c4, 5, 6, 7, 9, and 10 cluster cells have different tendencies.

D. MRG15L replaces MRG15S downregulates its transcriptional activation of *Mki67* on the PC1 axis of transcript expression, where *Cdkn2a* (*p16*) decreases with the axis, while *Mki67* decreases, as well as the expression of other proteins such as Top2a, Birc5, H2afz, Sqstm1, Grina, and Kdm5b.

E. The up-regulation or down-regulation of RNA quantity (>80 and >200) in c10 cell clusters is also significantly greater than other groups (<20, <50).

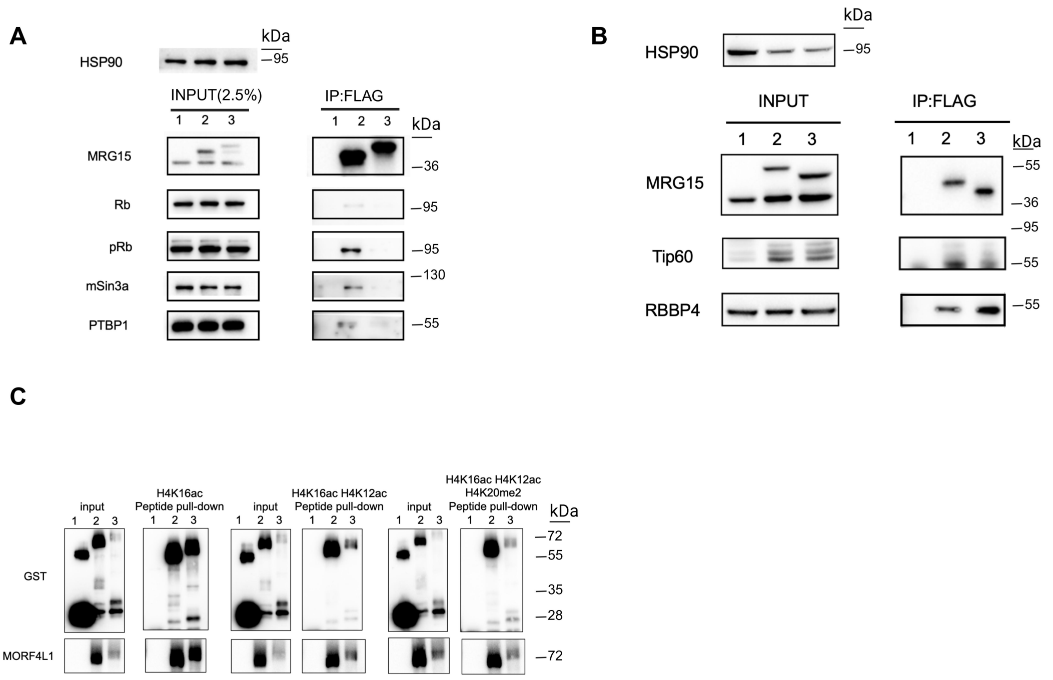

Supplementary Fig. 3: MRG15L and MRG15S interaction protein analysis.

A. GST-PULLDOWN experiment of MRG15L and MRG15S, prokaryotic purified GST (1), GST-MRG15S (2), and GST-MRG15L (3) were pulled down with 293T cell lysates. The binding ability of MRG15S to Rb, pRB, mSin3a, and PTBP1 previously reported is superior to MRG15L.

B. Transfection of PCMV-3tag6 (1), PCMV-3tag6-MRG15L (2), and PCMV-3tag6-MRG15S (3) followed by M2-antiFLAG bead CO-IP 24 hours later, the binding ability of MRG15L to Tip60 is slightly stronger than MRG15S.

C. Biotinylated H4K16ac, H4K12ac H4K16ac, and H4K12ac H4K16ac H4K20me2 peptides and prokaryotic purified GST (1), GST-MRG15S (2), and GST-MRG15L (3) were used for biotin affinity purification. The binding ability of MRG15S to H4 as a flowering protein is stronger than MRG15L.

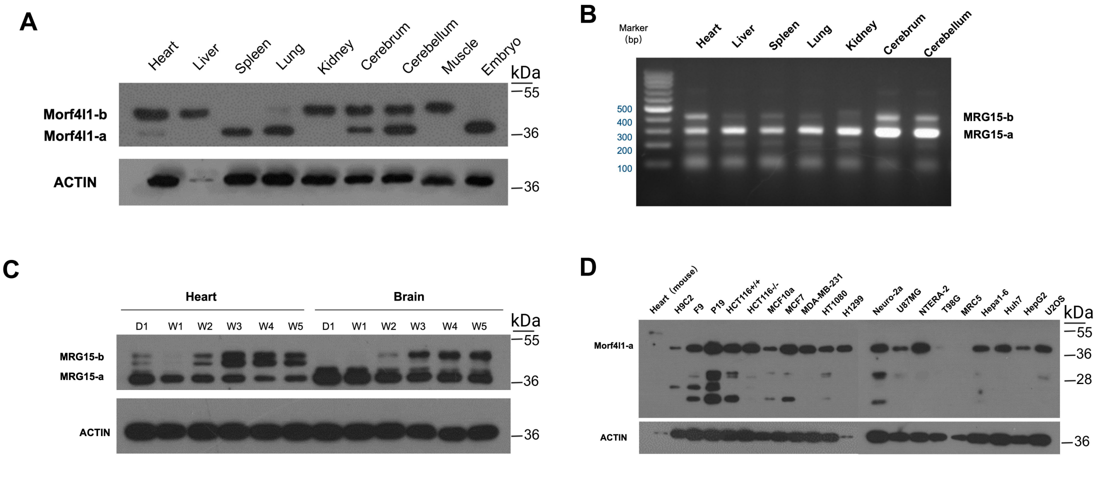

Supplementary Fig. 4: Specificity of MRG15 L/S tissue and development.

A. In mouse tissues, the expression levels of MRG15L/S variant proteins are distinct. The expression level of MRG15L is higher in liver, kidney, and muscle, while MRG15S is higher in spleen, lung, and embryonic tissues. MRG15L/S is expressed in both the cerebrum and cerebellum.

B. In mouse tissue mRNA, the expression level of *MRG15L* around 400bp is higher in the heart, cerebrum, and cerebellum.

C. The expression level of MRG15L/S variant proteins during postnatal development in mouse heart and brain tissues: MRG15L gradually increases in the heart and brain from D1, W1, W2, W3, W4 to W5, while MRG15S slowly decreases.

D. The expression status of MRG15S/L cell line in our laboratory showed that MRG15 existed in the form of S variant in almost all proliferating cells, while the control group consisted of mouse myocardial tissue

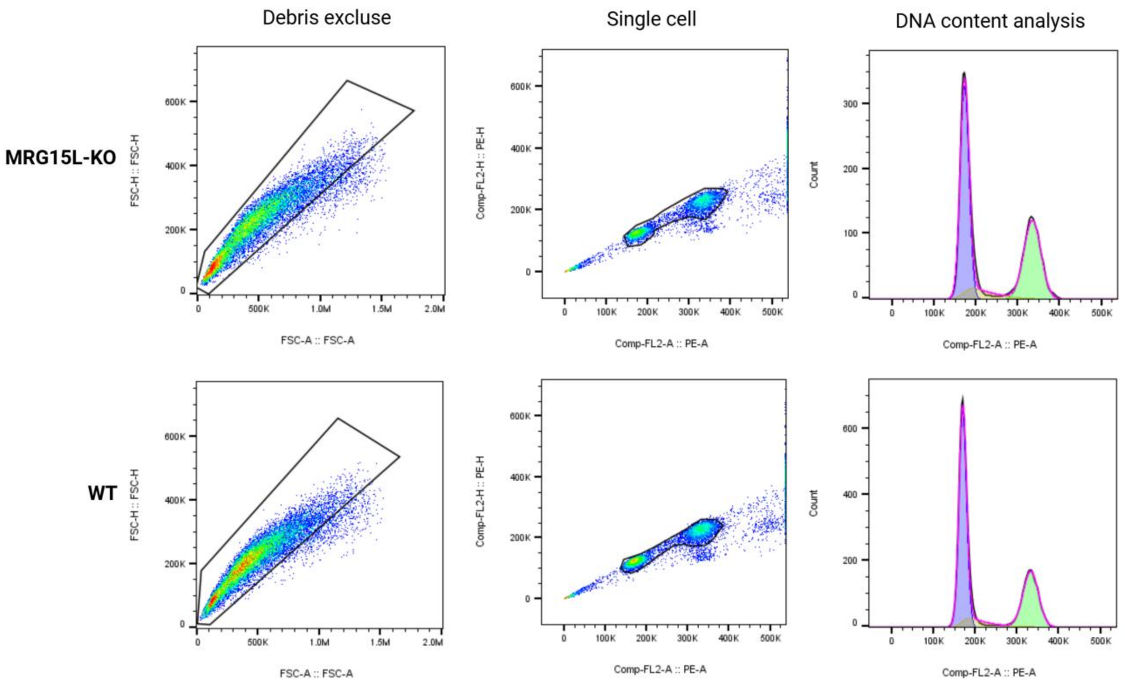

Supplementary Fig. 5: Flow cytometry gating strategy for DNA content analysis.

The gating includes debris exclusion (FSC-A vs FSC-H), singlet discrimination (PE-A height vs area), and PI-based DNA content analysis. In the DNA histogram, purple, yellow, and green regions represent cells in G1, S, and G2/M phases, respectively.

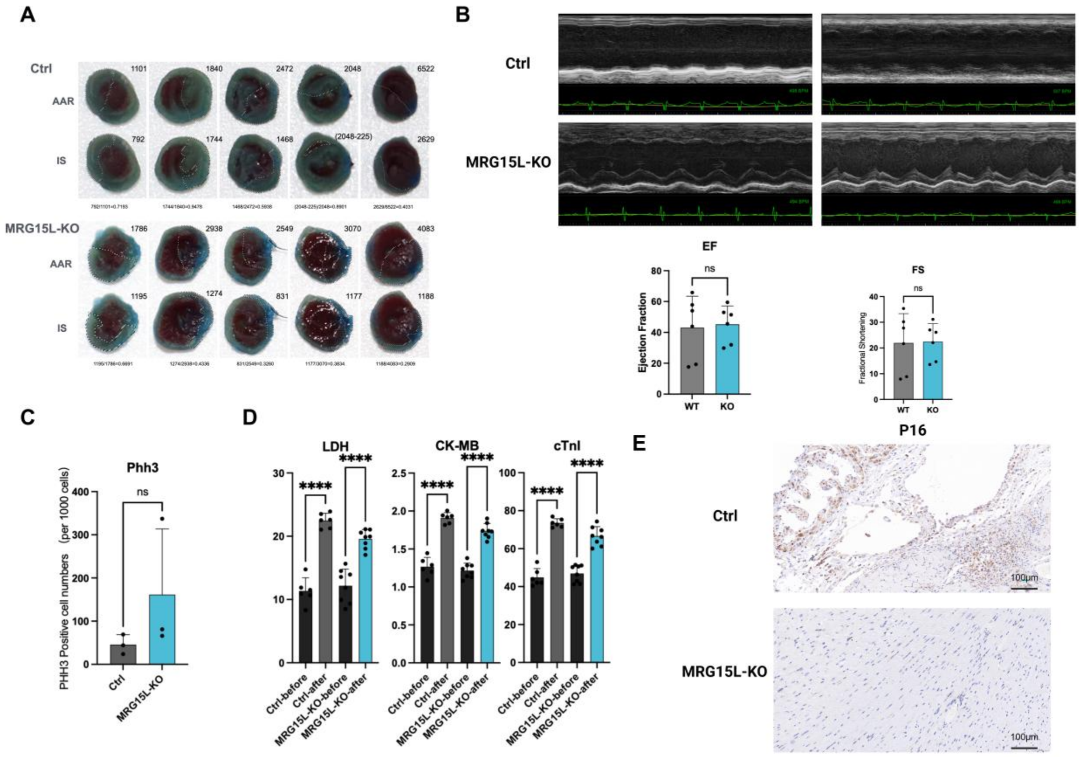

Supplementary Fig. 6: Myocardial ejection function after ischemia-reperfusion.

A. Quantification of left ventricular and infarct areas in control vs. MRG15L-KO groups. Areas were measured using ImageJ software (n=5 mice/group).

B. Representative M-mode tracings one week after myocardial ischemia-reperfusion injury in wild-type (Ctrl) and MRG15L-KO mice. After myocardial ischemia-reperfusion, the ES and FS functions of the myocardium after MRG15L knockout were slightly better than those of the control group (n=6 mice/group).Data represent mean ± SEM.

C. Quantitative analysis of PHH3+ cardiomyocytes in myocardial sections.No statistically significant difference was observed between groups (n=3 mice/group).Data represent mean ± SEM.

D. Serum levels of LDH, CK-MB, and cTnI pre- vs. post-myocardial ischemia-reperfusion injury (IRI). Significant elevation in all biomarkers post-IRI (n ⩾ 6 mice/group, data represent mean ± SEM.****p<0.0001 vs. baseline).

E. Representative P16 immunohistochemical staining in infarct border zones. MRG15L-KO mice exhibited reduced senescence compared to controls.

Supplementary Table 1. Primers for RT-qPCR and constructions

**Primers for cloning**	
MRG15-F	ATTAAGGATCCATGGCGCCCAAGCAGG
MRG15-R	TATATGTCGACCACGGCTTTCCGGTGG
Cdk1 promoter-F-(MluI)	ATTACGCGT GTTTCTTCCACCTCCTTCCCG
Cdk1 promoter-R-(XhoI)	AATCTCGAG CTAGACACGACCCTGACC
**Primers for RT-qPCR**	
q-mP16ink4a-F	CGCAGGTTCTTGGTCACTGT
q-mP16ink4a-R	TGTTCACGAAAGCCAGAGCG
q-mp21Waf1/Cip1-F	CCTGGTGATGTCCGACCTG
q-mp21Waf1/Cip1-R	CCATGAGCGCATCGCAATC
q-mGapdh-F	AGGTCGGTGTGAACGGATTTG
q-mGapdh-R	TGTAGACCATGTAGTTGAGGTCA
q-mPclaf-F	ACCAAAGCAAACTACGTTCCA
q-mPclaf-R	TTTTCCCGACGAACTTGAAGAA
q-mBirc5-F	GAGGCTGGCTTCATCCACTG
q-mBirc5-R	CTTTTTGCTTGTTGTTGGTCTCC
q-mTop2a-F	CAACTGGAACATATACTGCTCCG
q-mTop2a-R	GGGTCCCTTTGTTTGTTATCAGC
q-mMki67-F	ATCATTGACCGCTCCTTTAGGT
q-mMki67-R	GCTCGCCTTGATGGTTCCT
q-mHmgb2-F	GCTCGTTATGACAGGGAGATG
q-mHmgb2-R	TTGCCCTTGGCACGGTATG
q-mSmc2-F	GGCTGGGATTACCAAAGCCTC
q-mSmc2-R	CACCAATAACCACCTGTCTTGT
q-mH2afz-F	CCAAGACAAAGGCGGTTTCC
q-mH2afz-R	TTTCAGGTGTCGATGAATACGG
q-mCdca8-F	AAAAGCGAAAGGTAATCGAGGT
q-mCdca8-R	TGCAGATCGAAGATTCTTATGGC
q-mUbe2c-F	GAGTCAGACAACCTGTTCAAGTG
q-mUbe2c-R	TCTAGGGAGAGTTTGTACCTCAG
q-mCks2-F	TCGATGAGCACTACGAGTACC
q-mCks2-R	CCATCCTAGACTCTGTTGGACAC
q-mPrc1-F	AACTCACCTCCGGGAAATATGG
q-mPrc1-R	GGATATGCTTTTGAGCAGCCT
q-mTk1-F	AGTGCCTGGTCATCAAGTATGC
q-mTk1-R	GCTGCCACAATTACTGTCTTGC
q-mSmc4-F	AACTTCAAGTCCTATGCTGGAGA
q-mSmc4-R	TTGTGCTCGATAGCCAAACAC
q-mCks1b-F	TATTCGGACAAATACGACGACG

q-mCks1b-R	GGTTCCTCCATTCAGATTCAGAC
q-mStmn1-F	TCTGTCCCCGATTTCCCCC
q-mStmn1-R	AGCTGCTTCAAGACTTCCGC
q-mMxra8-F	GCGCCGACTTGTGGATATGTA
q-mMxra8-R	GCCGTCGTGGAAAGCAGAA
q-mCdkn2a-F	CGCAGGTTCTTGGTCACTGT
q-mCdkn2a-R	TGTTCACGAAAGCCAGAGCG
q-mDdit3-F	CTGGAAGCCTGGTATGAGGAT
q-mDdit3-R	CAGGGTCAAGAGTAGTGAAGGT
q-mBgn-F	TGCCATGTGTCCTTTCGGTT
q-mBgn-R	CAGGTCTAGCAGTGTGGTGTC
q-mItga11-F	TGCCCCAATGGAAACCAATG
q-mItga11-R	CACTCGTGCGACCAGAGAG
q-mTmem150a-F	GGTCCTACAATGAATCCTGCTC
q-mTmem150a-R	GGCCACCATAACAGCACCC
q-mSqstm1-F	AGGATGGGGACTTGGTTGC
q-mSqstm1-R	TCACAGATCACATTGGGGTGC
q-mKdm5b-F	CTGGGAAGAGTTCGCGGAC
q-mKdm5b-R	CGCGGGGTGAAATGAAGTTTAT
q-mGrina-F	CAAGCCCCTATGCCTCCCTAT
q-mGrina-R	GGCCCTTGAGGGTAACCAC
q-mHist1H2bc-F	GAGGAGCAGACCTGACATCG
q-mHist1H2bc-R	CACTGTCTTGAGGTTACAGCAT
q-mCcnd2-F	GAGTGGGAACTGGTAGTGTTG
q-mCcnd2-R	CGCACAGAGCGATGAAGGT
q-mYpel3-F	GCGGATTTCAAAGCCCAAGAC
q-mYpel3-R	TGACTGCCCTGGAAGGACTT
q-mMxd4-F	ATGGAGCTGAACTCTCTGCTG
q-mMxd4-R	GTGAAGACCTGTTGTTCGGGG

Supplementary Table 2. siRNA information

Gene	Sequence (5′→ 3′)
Human MRG15-1	Sense: GCUGAAAUUCUUGCAGAUCAUTT
	Antisense:
Human MRG15-2	Sense: CGGAGAGCAGAGUACUCAAAUTT
	Antisense:
Human MRG15-3	Sense: GUGUGUAAAGGUUGCCAUAAATT
	Antisense:

Supplementary Table 3. Antibody information in this study

Antibody	Brand	Apply
Mouse anti-Actin	1:5000, Santa Cruz Biotechnology, Sc-47778	WB
Rabbit anti-HSP90α	1:3000, Enzo, 11081317	WB
Rabbit anti-MRG15	1:2000, Cell Signaling Technology, 14098	WB
Rabbit anti-p16	1:4000, abcam, ab211542	WB
Rabbit anti-FLAG	1:1000, MBL, PM020	WB
Mouse anti-FLAG	1:10000, Sigma, SLBF1225	WB
Mouse anti-GST	1:3000, Santa Cruz Biotechnology, sc-138	WB
Mouse anti-MYC	1:2000, MBL, M047-3	WB
Mouse anti-Rb	1:500, Santa Cruz Biotechnology, sc-102	WB
Rabbit anti-Phospho-Rb	1:2000, Cell Signaling Technology, 8516	WB
Rabbit anti-mSin3a	1:500, Santa Cruz Biotechnology, sc-994	WB
Rabbit anti-HDAC1	1:1000, Cell Signaling Technology, 2062	WB
Mouse anti-CRISPR-Cas9	1:2000, abcam, ab191468	WB
Rabbit anti-p16	1:500, abcam, ab51243	IHC
Mouse anti-PHH3	1:200,Proteintech,66863-1-IG	IHC

Fig 2H-P16

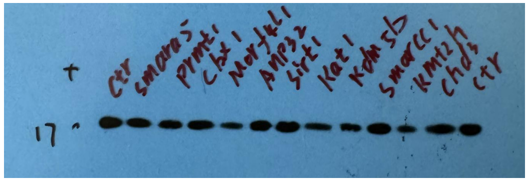

Fig 2H-Actin

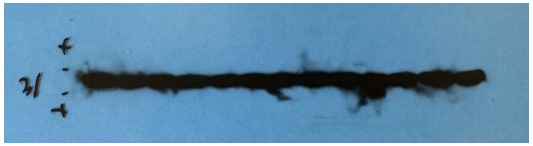

Figure 2J-Actin P16

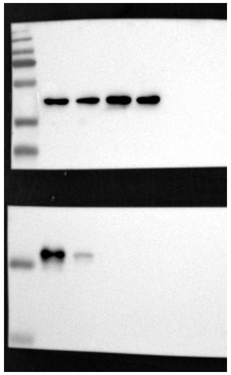

Figure 2J-MRG15

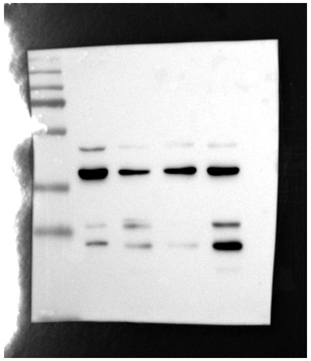

Fig 3A-Actin P16

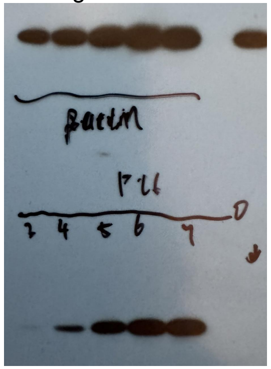

Fig 3A-MRG15

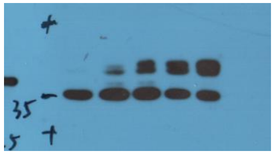

Fig 3B-Actin MRG15 P16

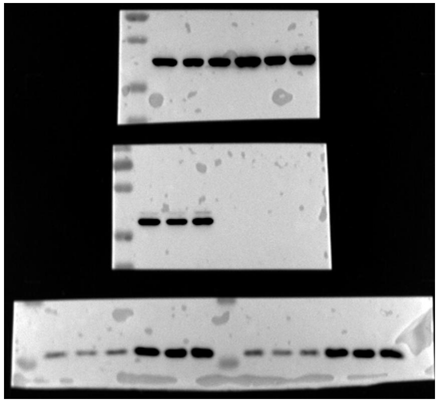

Figure 3D-Actin

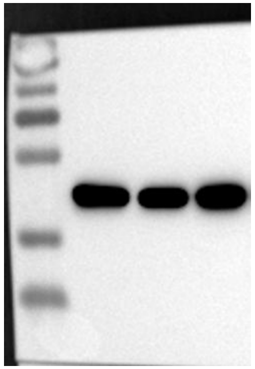

Figure 3D-MRG15 P16

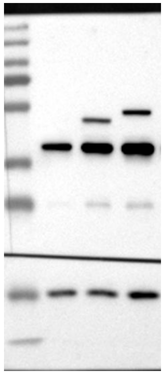

Figure 3E-Actin P16

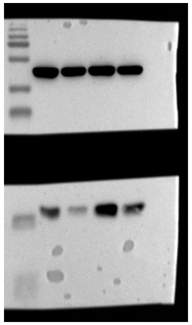

Figure 3E-MRG15

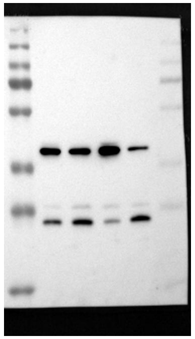

Figure 4C-Input

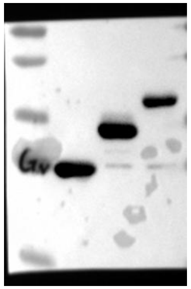

Figure 4C-pulldown

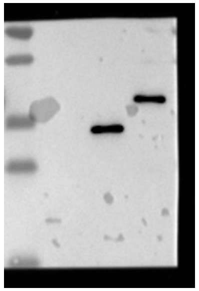

Figure 4D

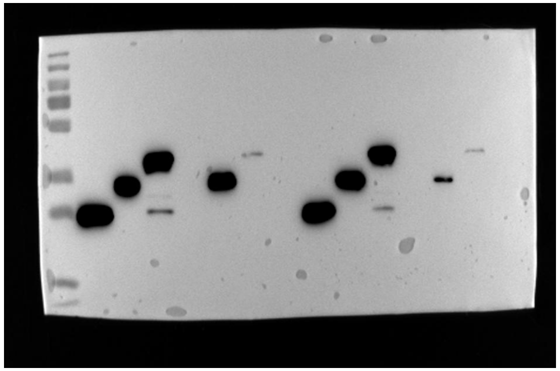

Supplementary Fig.1B-Actin

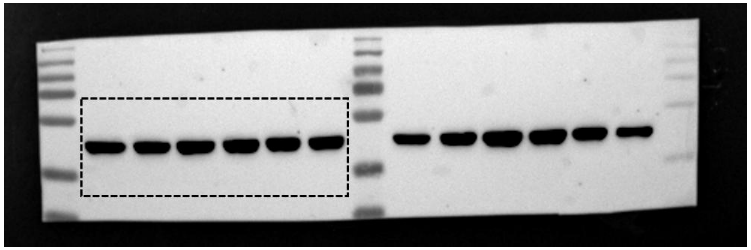

Supplementary Fig.1B-P16

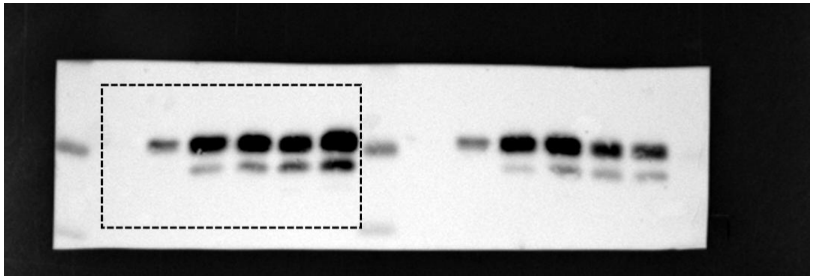

Supplementary Fig.1E

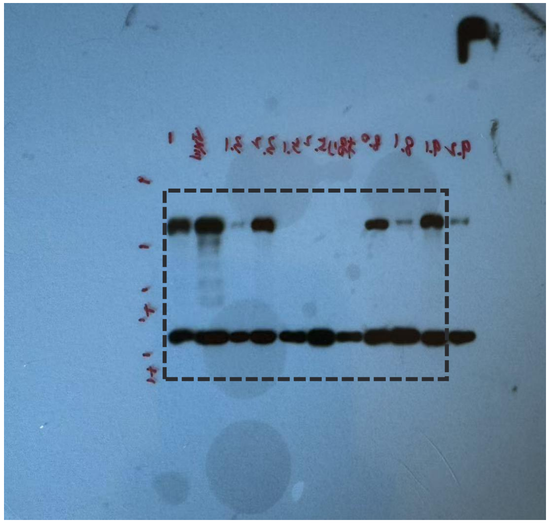

Supplementary Fig.3A-(INPUT) PTBP1

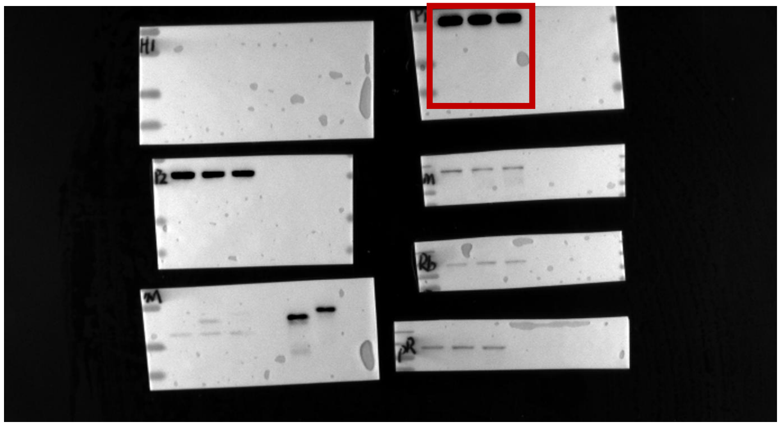

Supplementary Fig.3A-(INPUT) HSP90

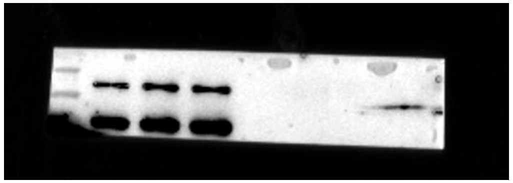

Supplementary Fig.3A-(INPUT IP) MRG15 (INPUT) mSin3a

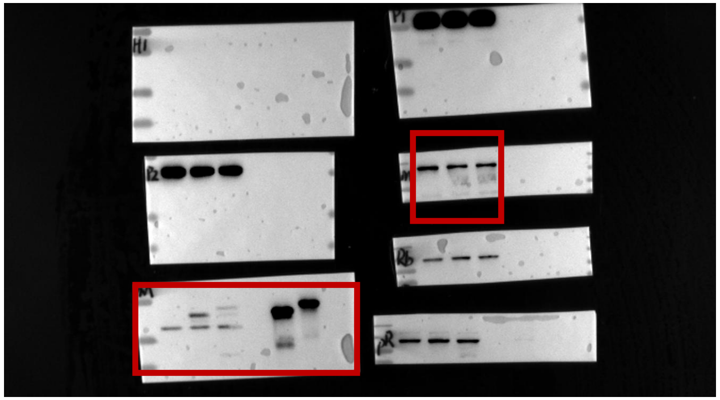

Supplementary Fig.3A-(INPUT) Rb (INPUT) pRb

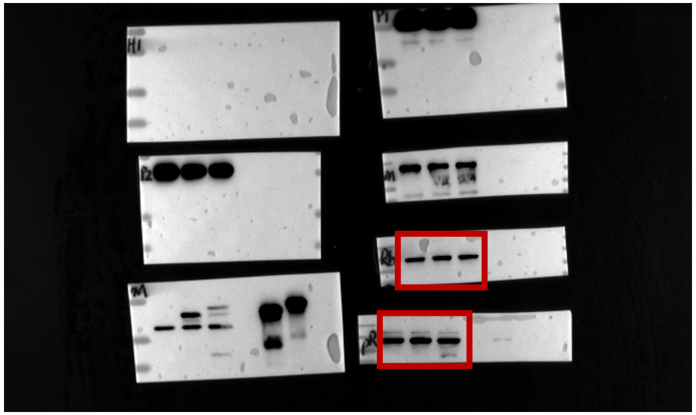

Supplementary Fig.3A-(IP) PTBP1 mSin3a Rb pRb

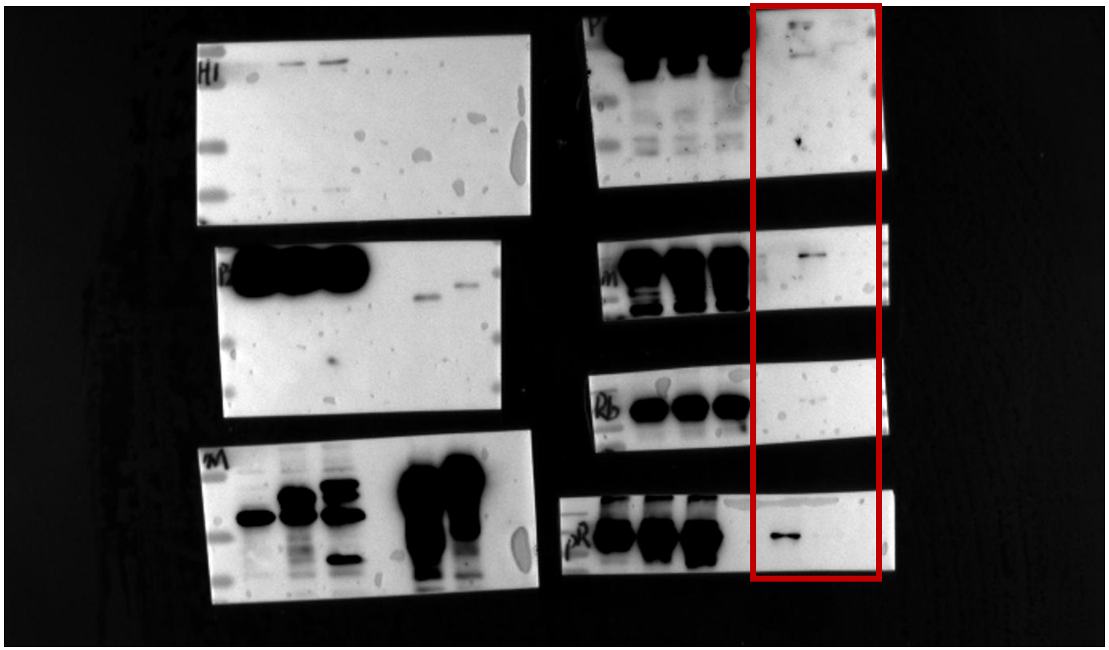

Supplementary Fig.3B-HSP90

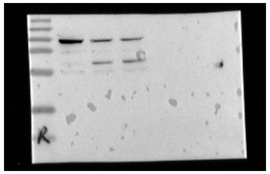

Supplementary Fig.3B-(INPUT) MRG15

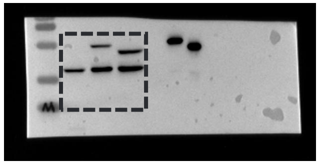

Supplementary Fig.3B-(IP) MRG15

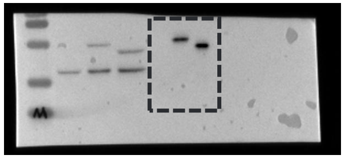

Supplementary Fig.3B-(INPUT) RBBP4

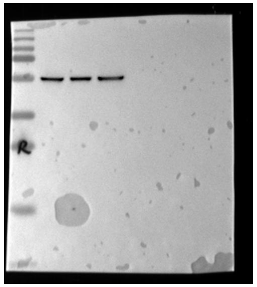

Supplementary Fig.3B-(IP) RBBP4

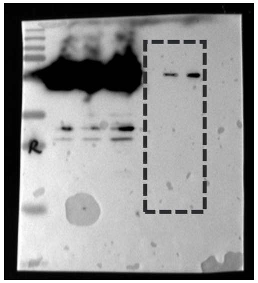

Supplementary Fig.3B-(INPUT) Tip60

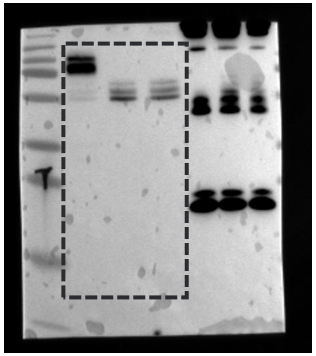

Supplementary Fig.3B-(IP) Tip60

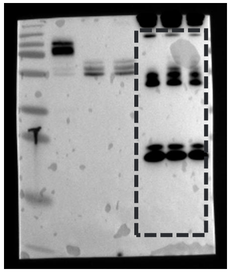

Supplementary Fig.3C GST input+pulldown

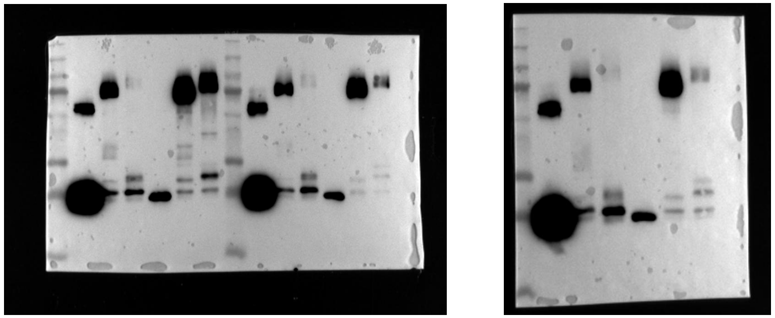

Supplementary Fig.3C MRG15 input+pulldown

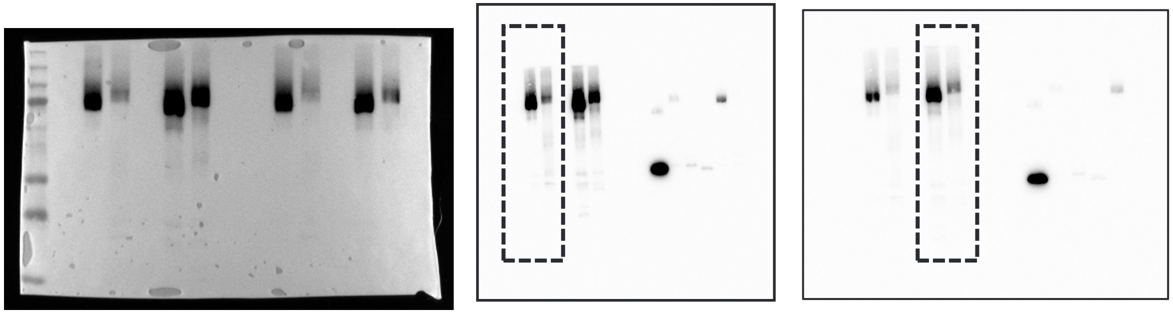

Supplementary Fig.4A-MRG15(Morf4l1)

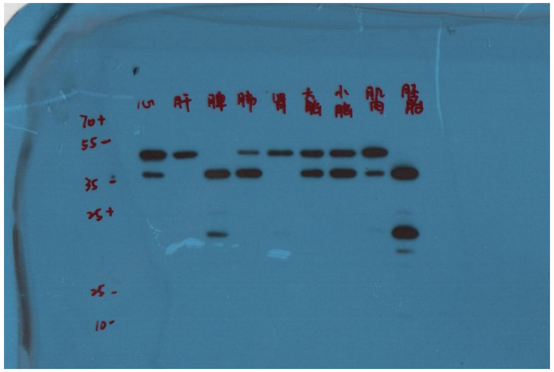

Supplementary Fig.4A-ACTIN

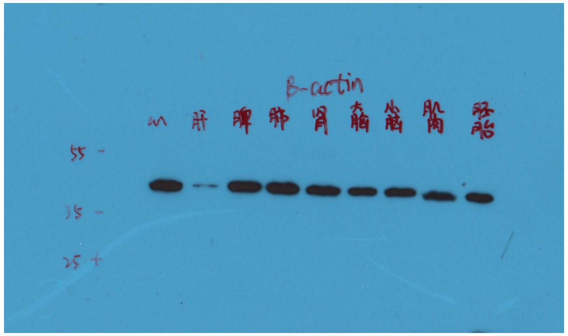

Supplementary Fig.4C

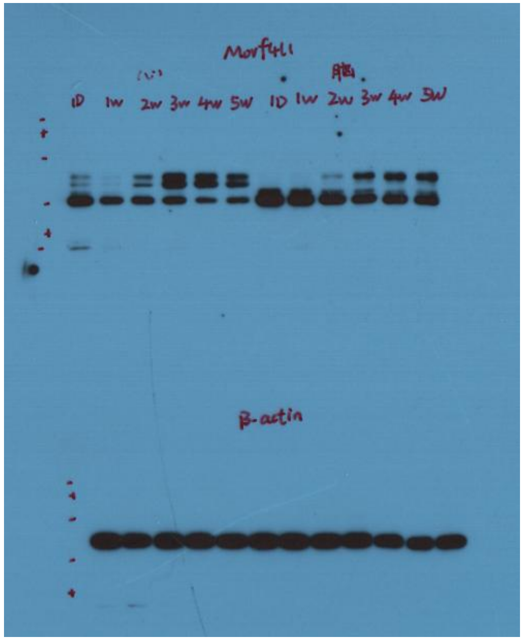

Supplementary Fig.4D

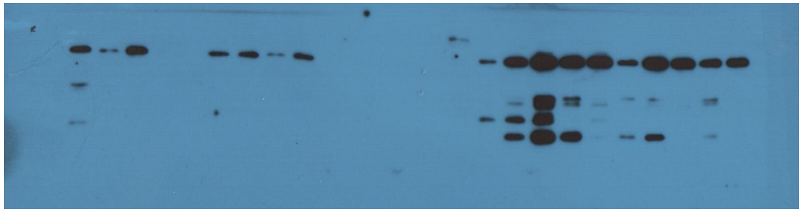

Supplementary Fig. 7: Original blot images for all the experiments presented in the manuscript.
The cropped regions used in the main figures are indicated by black dashed boxes or red rectangles.
