# Supplementary material for: MRG15 alternative splicing regulates CDK1 transcriptional activity in mouse cell senescence and myocardial regeneration
Source: Commun Biol. 2025 Jun 7;8:895. doi: 10.1038/s42003-025-08309-z (PMC12145428; doi:10.1038/s42003-025-08309-z)
Supplement: Supplementary file 2 — Description of Additional Supplementary Materials [file 42003_2025_8309_MOESM2_ESM.pdf]

Description of Additional Supplementary Files

File name: Supplementary Data 1

Description: The full list of gRNA sequences in the study

File name: Supplementary Data 2

Description: The source data behind the graphs in the paper
