# Supplementary material for: MRG15 alternative splicing regulates CDK1 transcriptional activity in mouse cell senescence and myocardial regeneration
Source: Commun Biol. 2025 Jun 7;8:895. doi: 10.1038/s42003-025-08309-z (PMC12145428; doi:10.1038/s42003-025-08309-z)
Supplement: Supplementary file 5 — Reporting Summary [file 42003_2025_8309_MOESM5_ESM.pdf]

nature portfolio

Corresponding author(s): Professor Ye Zhang

Last updated by author(s): May 20, 2025

Reporting Summary

Nature Portfolio wishes to improve the reproducibility of the work that we publish. This form provides structure for consistency and transparency in reporting. For further information on Nature Portfolio policies, see our Editorial Policies and the Editorial Policy Checklist.

Statistics

For all statistical analyses, confirm that the following items are present in the figure legend, table legend, main text, or Methods section.

n/a	Confirmed	
☐	☒	The exact sample size (*n*) for each experimental group/condition, given as a discrete number and unit of measurement
☐	☒	A statement on whether measurements were taken from distinct samples or whether the same sample was measured repeatedly
☐	☒	The statistical test(s) used AND whether they are one- or two-sided<br>*Only common tests should be described solely by name; describe more complex techniques in the Methods section.*
☒	☐	A description of all covariates tested
☐	☒	A description of any assumptions or corrections, such as tests of normality and adjustment for multiple comparisons
☐	☒	A full description of the statistical parameters including central tendency (e.g. means) or other basic estimates (e.g. regression coefficient) AND variation (e.g. standard deviation) or associated estimates of uncertainty (e.g. confidence intervals)
☐	☒	For null hypothesis testing, the test statistic (e.g. *F*, *t*, *r*) with confidence intervals, effect sizes, degrees of freedom and *P* value noted<br>*Give P values as exact values whenever suitable.*
☒	☐	For Bayesian analysis, information on the choice of priors and Markov chain Monte Carlo settings
☒	☐	For hierarchical and complex designs, identification of the appropriate level for tests and full reporting of outcomes
☒	☐	Estimates of effect sizes (e.g. Cohen's *d*, Pearson's *r*), indicating how they were calculated

Our web collection on statistics for biologists contains articles on many of the points above.

Software and code

Policy information about availability of computer code

Data collection	Data collection was performed using Bio-Rad CFX 100 for qPCR and standard single-cell sequencing platforms for transcriptome analysis.
Data analysis	Data analysis was performed using GraphPad Prism 9 for statistical analysis and FlowJo for flow cytometry data processing.

For manuscripts utilizing custom algorithms or software that are central to the research but not yet described in published literature, software must be made available to editors and reviewers. We strongly encourage code deposition in a community repository (e.g. GitHub). See the Nature Portfolio guidelines for submitting code & software for further information.

Data

Policy information about availability of data

All manuscripts must include a data availability statement. This statement should provide the following information, where applicable:

- Accession codes, unique identifiers, or web links for publicly available datasets
- A description of any restrictions on data availability
- For clinical datasets or third party data, please ensure that the statement adheres to our policy

The datasets generated and analyzed during the current study are available from the corresponding author upon reasonable request.

Research involving human participants, their data, or biological material

Policy information about studies with human participants or human data. See also policy information about sex, gender (identity/presentation), and sexual orientation and race, ethnicity and racism.

Reporting on sex and gender	Not applicable. This study does not involve human participants or human data.
Reporting on race, ethnicity, or other socially relevant groupings	Not applicable. This study does not involve human participants or socially relevant groupings.
Population characteristics	Not applicable. This study involves mouse models and does not involve human participants.
Recruitment	Not applicable. No human participants were recruited for this study.
Ethics oversight	All animal experiments were conducted in accordance with the ethical guidelines approved by the Laboratory Animal Welfare and Ethics Committee of Beijing Medconn Biotechnology Co., Ltd., under approval number MDKN-2024-049.

Note that full information on the approval of the study protocol must also be provided in the manuscript.

Field-specific reporting

Please select the one below that is the best fit for your research. If you are not sure, read the appropriate sections before making your selection.

☒ Life sciences ☐ Behavioural & social sciences ☐ Ecological, evolutionary & environmental sciences

For a reference copy of the document with all sections, see nature.com/documents/nr-reporting-summary-flat.pdf

Life sciences study design

All studies must disclose on these points even when the disclosure is negative.

Sample size	Sample sizes were determined based on standard experimental practices for mouse models, with at least n=3 animals per group to ensure statistical power and reproducibility.
Data exclusions	No data were excluded from the analyses.
Replication	All experiments were performed with biological replicates, and all findings were successfully replicated to ensure reproducibility.
Randomization	Randomization was not applicable as allocation to experimental groups (e.g., wild-type vs. gene knockout mice) was determined by genotype.
Blinding	Investigators were blinded to group allocation during data collection and analysis to minimize bias.

Reporting for specific materials, systems and methods

We require information from authors about some types of materials, experimental systems and methods used in many studies. Here, indicate whether each material, system or method listed is relevant to your study. If you are not sure if a list item applies to your research, read the appropriate section before selecting a response.

Materials & experimental systems

n/a	Involved in the study	
☐	☒	Antibodies
☐	☒	Eukaryotic cell lines
☒	☐	Palaeontology and archaeology
☐	☒	Animals and other organisms
☒	☐	Clinical data
☒	☐	Dual use research of concern
☒	☐	Plants

Methods

n/a	Involved in the study	
☒	☐	ChIP-seq
☐	☒	Flow cytometry
☒	☐	MRI-based neuroimaging

Antibodies

Antibodies used	The following antibodies were used in this study:- Mouse anti-Actin (1:5000, Santa Cruz Biotechnology, catalog no. sc-47778) for Western blot (WB).- Rabbit anti-HSP90α (1:3000, Enzo, catalog no. 11081317) for WB.- Rabbit anti-MRG15 (1:2000, Cell Signaling Technology, catalog no. 14098) for WB.- Rabbit anti-p16 (1:4000, Abcam, catalog no. ab211542) for WB.- Rabbit anti-FLAG (1:1000,

	MBL, catalog no. PM020) for WB.- Mouse anti-FLAG (1:1000, Sigma, catalog no. SLBF1225) for WB.- Mouse anti-GST (1:3000, Santa Cruz Biotechnology, catalog no. sc-138) for WB.- Mouse anti-MYC (1:2000, MBL, catalog no. M047-3) for WB.- Mouse anti-Rb (1:500, Santa Cruz Biotechnology, catalog no. sc-102) for WB.- Rabbit anti-Phospho-Rb (1:2000, Cell Signaling Technology, catalog no. 8516) for WB.- Rabbit anti-mSin3a (1:500, Santa Cruz Biotechnology, catalog no. sc-994) for WB.- Rabbit anti-HDAC1 (1:1000, Cell Signaling Technology, catalog no. 2062) for WB.- Mouse anti-CRISPR-Cas9 (1:2000, Abcam, catalog no. ab191468) for WB.- Mouse anti-PHH3 (1:200, Proteintech, catalog no. 66863-1-IG) for immunohistochemistry (IHC).
Validation	All antibodies used in this study were validated for their respective applications and species by the manufacturers. Validation data, including specificity and use in Western blot (WB) and immunohistochemistry (IHC), are available on the manufacturers' websites. Each antibody has been cited in prior peer-reviewed publications supporting its use in similar experimental settings.

Eukaryotic cell lines

Policy information about cell lines and Sex and Gender in Research

Cell line source(s)	The MEF (mouse embryonic fibroblast) cell lines used in this study were derived from C57BL/6J mice and established in our laboratory. The HEK293T cell lines were obtained from the cell line repository maintained by our research group and are human embryonic kidney cells with the SV40 T-antigen transformation.
Authentication	The HEK293T cell lines were not authenticated after retrieval from the research group repository. The MEF cell lines were derived directly from primary tissues of C57BL/6J mice and were not further authenticated.
Mycoplasma contamination	The HEK293T and MEF cell lines were not tested for mycoplasma contamination in this study.
Commonly misidentified lines (See ICLAC register)	The HEK293T and MEF cell lines used in this study are not listed as commonly misidentified cell lines in the ICLAC register.

Animals and other research organisms

Policy information about studies involving animals; ARRIVE guidelines recommended for reporting animal research, and Sex and Gender in Research

Laboratory animals	C57BL/6J mice were used in this study, including wild-type and MRG15L knockout male mice. The mice were 8-12 weeks old at the time of experiments.
Wild animals	This study did not involve wild animals.
Reporting on sex	Only male mice were used in this study to minimize variability introduced by hormonal cycles. No sex-based analysis was performed as female mice were not included in the experimental design.
Field-collected samples	This study did not involve field-collected samples.
Ethics oversight	All animal experiments were conducted in accordance with the ethical guidelines approved by the Laboratory Animal Welfare and Ethics Committee of Beijing Medconn Biotechnology Co., Ltd., under approval number MDKN-2024-049.

Note that full information on the approval of the study protocol must also be provided in the manuscript.

Plants

Seed stocks	This study did not involve the use of any seed stocks or plant material.
Novel plant genotypes	This study did not involve the generation or use of any novel plant genotypes.
Authentication	This study did not involve the use of any seed stocks or novel plant genotypes; therefore, no authentication procedures were required.

Flow Cytometry

Plots

Confirm that:

☒ The axis labels state the marker and fluorochrome used (e.g. CD4-FITC).

☒ The axis scales are clearly visible. Include numbers along axes only for bottom left plot of group (a 'group' is an analysis of identical markers).

☒ All plots are contour plots with outliers or pseudocolor plots.

☒ A numerical value for number of cells or percentage (with statistics) is provided.

Methodology

Sample preparation	MEF cells were fixed in 70% ethanol at 4°C for at least 30 minutes, stained with propidium iodide (PI), and treated with RNaseA to remove RNA. Cells were filtered through a 400-mesh strainer before flow cytometry analysis.
Instrument	Flow cytometry data were collected using a Beckman Coulter CytoFlex flow cytometer.
Software	Data were analyzed using FlowJo software (version 10.8).
Cell population abundance	The proportions of cells in G1, S, and G2/M phases were quantified using PI fluorescence for DNA content analysis. Results are reported as percentages of the total cell population.
Gating strategy	The gating includes debris exclusion (FSC-A vs FSC-H), singlet discrimination (PE-A height vs area), and PI-based DNA content analysis.

☒ Tick this box to confirm that a figure exemplifying the gating strategy is provided in the Supplementary Information.
